# The implementation of guidelines in palliative care – a scoping review

**DOI:** 10.1186/s12904-025-01729-y

**Published:** 2025-04-11

**Authors:** Alexandra Ernst, Franziska Schade, Stephanie Stiel, Katharina van Baal, Franziska A. Herbst

**Affiliations:** Institute for General Practice and Palliative Care, Hannover Medical School, Carl-Neuberg-Strasse 1, 30625 Hannover, Germany

**Keywords:** Palliative care, Guidelines, Literature review, Implementation, End-of-life care, Health services research

## Abstract

**Background:**

Guidelines are essential tools for ensuring high-quality healthcare. However, discrepancies exist between their availability and practical implementation. In the context of palliative care, the extent to which guidelines are implemented and the barriers and facilitators that influence this process remain unknown.

**Aim:**

The present study aimed at systematically reviewing the international literature on the implementation of palliative care guidelines to evaluate factors that support or hinder implementation of palliative care guidelines globally.

**Method:**

A scoping review was conducted following the methodological approach of Arksey and O’Malley (2005). After the formulation of research questions and development of a search string, relevant studies investigating the implementation of guidelines were identified and retrieved from the databases CINAHL, LIVIO, PubMed and Web of Science Core Collection on 4 January 2024. Two researchers independently selected articles for inclusion, employing a blinded process with predefined inclusion and exclusion criteria. The results were subsequently categorised deductively by the same researchers using Petermann’s (2014) taxonomy of implementation outcomes. The results were summarised and presented in tabular form.

**Results:**

The search yielded 2,086 records, of which 1,252 were included in the title and abstract screening. Subsequently, 113 full-text articles were reviewed for eligibility, resulting in 29 articles deemed suitable for the final analysis. Six implementation outcomes were identified in the included literature: (1) acceptability (*n* = 15 articles), (2) adoption (*n* = 6 articles), (3) appropriateness (*n* = 9 articles), (4) feasibility (*n* = 9 articles), (5) fidelity/adherence (*n* = 14 articles) and (6) penetration (*n* = 14 articles). The majority of studies employed quantitative approaches (*n* = 22) and considered the perspective of healthcare professionals and their opinions regarding guideline implementation in palliative care. Only 4 articles considered patient related outcomes or the perspectives of the family caregivers. Ten articles reported on facilitators and barriers. Facilitators included healthcare professionals’ motivation and managerial support, while barriers primarily referred to time constraints and limited knowledge.

**Conclusions:**

Guideline implementation in palliative care is highly variable. Future research should aim at comprehensively analysing facilitators of and barriers to this process, considering diverse implementation outcomes. For these evaluations, mixed-method approaches are recommended.

**Supplementary Information:**

The online version contains supplementary material available at 10.1186/s12904-025-01729-y.

## Background

Guidelines play a pivotal role in modern healthcare, providing systematic summaries of the latest evidence on health-related topics. They serve as concise, reliable sources of information for healthcare providers and offer structured recommendations for clinical decision-making across a wide range of health conditions [1]. While guidelines aim to standardise care, they also accommodate individual patient circumstances, allowing healthcare professionals to deviate from the recommendations in exceptional cases [1, 2].

There is a well-documented discrepancy between the availability of guidelines and their actual implementation in clinical practice [3]. However, possibilities to reduce this discrepancy are becoming increasingly relevant in the field of guideline development. As an example, the UK National Institute for Health and Care Excellence (NICE) has included a mission to support guideline implementation in its 2021–2026 strategy, including activities such as improving access and developing implementation strategies [4]. This increased focus on planning the implementation of guidelines in order to promote their realisation in practice can also be observed in international literature. An updated scoping review by Peters et al. [5], supported by the Guideline International Network (G-I-N), showed a noticeable increase in the number of studies dealing with implementation planning approaches and using, for example, frameworks or involving stakeholders in implementation planning [5].

One essential strategy to enhance practical implementation is addressing potential user concerns during the guideline development process. Hence, the Association of Scientific Medical Societies in Germany (AWMF) emphasises the need for thorough preparatory work prior to guideline implementation, including barrier analysis and the establishment of robust implementation strategies [6, 7]. Expert groups tasked with guideline development are encouraged to consider facilitators and barriers at multiple levels, including patient factors (e.g., comorbidities), staff influences (e.g., time constraints), educational supports (e.g., interactive training) and organisational dynamics (e.g., local consensus processes) [7].

Germany has made significant strides in guideline development, particularly in the field of oncology. In 2008, the German Guideline Program in Oncology was initiated, supported by the German Cancer Aid, the German Cancer Society and the Association of the Scientific Medical Societies in Germany. The aim of this program was to develop guidelines with the highest level of evidence for all oncological diseases. As a result of this initiative, by 2019, guidelines were available for over 90% of all oncological conditions [8, 9].

The implementation of these oncological guidelines has been facilitated through their integration with certification processes, with hospitals demonstrating guideline adherence eligible for certification as oncology centres by the German Cancer Society. Implementation is assessed using quality indicators derived from the guidelines, which are also considered during certification audits [9]. Studies have demonstrated the positive impact of this certification system (and thus guideline-compliant care) for patient outcomes. For example, Schmitt et al. [10] demonstrated that patients treated for the 11 most common cancer types in certified cancer centres had significantly longer overall survival compared to those treated in non-certified facilities. Similarly, another study found that adherence to guideline-based treatment for breast cancer patients was significantly associated with improved relapse-free and overall survival rates [11].

The S3 guideline on palliative care for patients with incurable cancer was developed as part of the German Guideline Program in Oncology and first introduced in 2015 [12], with a subsequent revision in 2019 [13]. This guideline provides comprehensive recommendations for palliative care, including 11 quality indicators and corresponding quality objectives. In contrast to other oncological guidelines, which focus on specific cancer entities, the S3 guideline on palliative care is broadly applicable to all oncology patients in the final stages of life who are receiving palliative care. Its primary aim is to improve symptom management and enhance the quality of palliative care provided to patients with incurable cancer [13].

The extent to which the S3 guideline on palliative care and its associated quality indicators have been implemented in palliative care wards in Germany remains unclear. Furthermore, little is known about the barriers and facilitators that influence the implementation process. These gaps in understanding form the subject of the research project ‘Quincie – Implementation of quality indicators from the S3 guideline on palliative care for patients with incurable cancer in the care of palliative wards’ [14]. As the first step in the project, the present scoping review aimed at providing an overview of the available evidence on the implementation of guidelines in palliative care. Given that palliative care guidelines have been established in other countries for a longer period (e.g., in the UK since 2004 [15]), the review drew on international literature.

## Methods

### Study aim

The scoping review aimed at systematically collecting and analysing the international literature on the implementation of palliative care guidelines to gain an overview of current evidence on guideline implementation processes. The review also sought to identify factors that either facilitate or hinder guideline implementation in palliative care settings. In contrast to intervention studies, the present review examined the broader context of palliative care guidelines and implementation research.

The following two research questions were addressed:

1. What is known from the international literature about guideline implementation within the field of palliative care?
2. What facilitators of and barriers to the implementation of palliative care guidelines have been identified?

### Reasons for choosing a scoping review approach

A scoping review approach was selected as the most suitable methodological approach to address the research questions as this approach offers the advantage of incorporating diverse study types and methodologies. Additionally, the scoping review approach allows for the inclusion of qualification theses and descriptive reports.

### Scoping review steps

The present scoping review was conducted in accordance with the methodological framework outlined by Arksey and O’Malley [16]. The process began with the formulation of research questions and the creation of a search string to identify the relevant literature. Subsequently, inclusion and exclusion criteria were defined based on the research questions, enabling two researchers to independently select articles through a blinded process. Eligible articles were then summarised and presented in tabular form. Finally, the results were summarised and reported using the Preferred Reporting Items for Systematic reviews and Meta-Analyses extension for Scoping Reviews (PRISMA-ScR) [17].

**Fig. 1:**
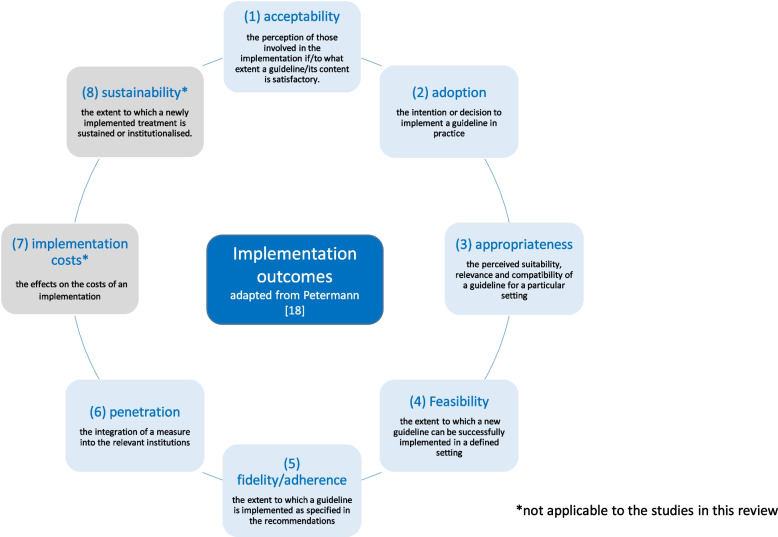
Implementation outcomes adopted from Petermann [18]

The results were organised following the taxonomy proposed by Petermann (see Fig. 1) [18], encompassing eight distinct implementation outcomes with corresponding assessment methods: (1) acceptability, (2) adoption, (3) appropriateness, (4) feasibility, (5) fidelity, (6) penetration, (7) cost and (8) sustainability [18]. Articles included in the review were deductively categorised and analysed by one researcher on the basis of these eight implementation outcomes. A second researcher verified the allocation of articles. Disagreements were resolved through discussion. Given that a single article could address multiple implementation outcomes, the assignment of more than one category was sometimes necessary. Due to its frequent use as a synonymous term, ‘adherence’ was added as a subcategory of fidelity, with both fidelity and adherence referring to the degree to which current practices aligned with guideline recommendations. Of note, while the taxonomy provided a valuable framework for categorising outcomes, some outcomes were inherently ambiguous, and the classification was therefore considered a guiding structure rather than a definitive categorisation [18].

### Inclusion criteria

#### Types of studies

The literature search was inclusive of all study designs that provided results relevant to the research questions. Conference abstracts and study protocols were excluded from the analysis. Although review articles were considered in order to contextualise the findings, they were not included in the charting of the results.

#### Time span

The German S3 guideline on palliative care [13] was first introduced in 2015 and revised in 2019. However, international guidelines in palliative care have been available for much longer. For example, the UK published its first guideline, ‘Improving supportive and palliative care for adults with cancer’, in 2004 [15]. Since the exact timelines for the implementation of international guidelines and corresponding implementation studies are unclear, no restrictions were placed on study publication date.

#### Languages

Studies published in English and German were included. English articles were selected to incorporate international research on guideline implementation. As the scoping review supported the German project ‘Quincie’, articles published in German were also included.

#### Databases

To maximise the inclusion of relevant articles and minimise the risk of omission, literature searches were conducted in five databases. Primary test searches led to the selection of the following databases: CINAHL, LIVIO, PubMed and Web of Science Core Collection. While Google Scholar was initially also included, subsequent test searches revealed no additional relevant results from this database, leading to its exclusion. The final search was conducted across all selected databases on 4 January 2024.

#### Search strategy

The search strategy was developed iteratively, beginning with test searches in PubMed and adjusted to optimise the relevant results. Once finalised, the PubMed strategy was adapted for the remaining databases. The core terms of the research questions (i.e., palliative care, implementation, guideline), along with suitable synonyms, were combined using Boolean operators (AND, OR). Studies focusing on paediatric palliative care were excluded using the ‘NOT’ operator, as separate guidelines exist for this population. The final search strategies are provided as Additional file 1.

#### Content-related inclusion and exclusion criteria

Articles were initially assessed according to formal criteria (i.e., language) to determine eligibility. Subsequently, a content-related screening process was conducted using a hierarchical approach, evaluating whether each article: (1) related to a palliative care setting, (2) addressed guidelines or guideline-based quality indicators and (3) explicitly analysed guideline implementation, including corresponding outcomes. Articles that failed to meet any of these criteria were excluded. Articles with no full text available were also excluded.

#### Guidelines

The review included studies with guidelines whose implementation in palliative care was analysed. This encompassed not only studies dealing with national palliative care guidelines, but also articles considering symptom-related and ethical guidelines.

## Results

### Study selection

**Fig. 2:**
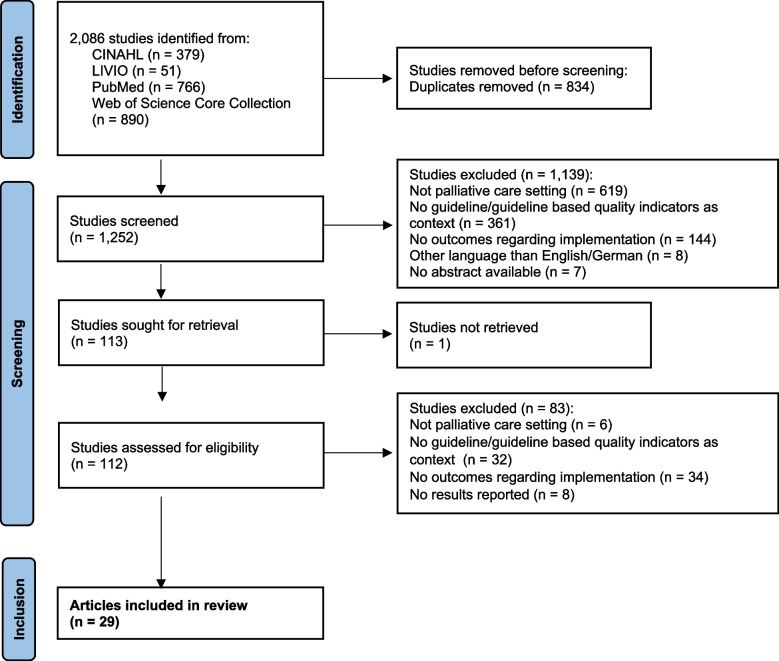
Study selection flow chart

The database search identified 2,086 relevant articles. All references, including abstracts, were imported into Endnote 20 (Clarivate, Philadelphia, USA) for reference management and duplicate removal. After duplicates were excluded, 1,252 articles proceeded to the title and abstract screening. Using the online tool rayyan [19], two researchers independently performed a blinded screening of titles and abstracts based on the predefined inclusion and exclusion criteria. During this process, rayyan was utilised solely as a screening tool; no AI features were used. Disagreements regarding inclusion or exclusion were resolved through discussion following the blinded screening. At this stage, 113 articles advanced to full-text screening (see Fig. 2 for a flow chart of the study selection procedure).

The full-text screening was conducted in Endnote by the same two authors using the same blinded approach. One study was excluded because the full text could not be retrieved. Disagreements were resolved through discussion. Ultimately, 29 articles met the inclusion criteria and were included in the final analysis.

### Characteristics of the included studies

The final set of articles comprised 29 original research papers published between 2001 and 2023. Among these, one study [20*] was categorised as a clinical audit report, and another [21*] as an evidence utilisation study. No review articles were identified. Geographically, the majority of studies originated from the USA (*n* = 7) [22*, 23*, 24*, 25*, 26*, 27*, 28*], followed by Australia (*n* = 4) [21*, 29*, 30*, 31*] and Germany (*n* = 4) [32*, 33*, 34*, 35*]. Additional studies were conducted in the UK [20*, 36*, 37*], Canada [38*, 39*], Denmark [40*, 41*], Belgium [42*], Sweden [43*], Norway [44*], Scotland [45*], Singapore [46*], the Netherlands [47*] and Switzerland [48*]. Regarding methodology, most studies employed quantitative approaches (*n* = 22) [20*, 21*, 22*, 23*, 24*, 25*, 26*, 27*, 28*, 29*, 30*, 32*, 33*, 34*, 35*, 37*, 39*, 40*, 42*, 44*, 45*, 48*]. Study participants were predominantly healthcare professionals (*n* = 19) [20*, 22*, 24*, 25*, 27*, 29*, 31*, 32*, 33*, 34*, 35*, 36*, 38*, 41*, 43*, 44*, 46*, 47*, 48*], while patients were also frequently included, often through retrospective chart analyses (*n* = 9) [23*, 24*, 37*, 38*, 39*, 40*, 42*, 45*, 46*]. Patient surveys (*n* = 3) [26, 28, 30] and surveys with families of deceased patients (*n* = 1) [47] were less common. In terms of guideline focus, most studies examined the implementation of national (*n* = 13) [21*, 22*, 23*, 24*, 26*, 28*, 33*, 34*, 41*, 43*, 44*, 46*, 47*], regional (*n* = 2) [31*, 35*] or international [36*] guidelines on palliative care. Specific types of guidelines included cancer pain guidelines (*n* = 3) [29*, 30*, 45*], symptom-specific guidelines (*n* = 2) [37*, 40*] and others, including legal/ethical guidelines [32*, 48*].

In relation to the second research question, 10 [20*, 21*, 31*, 33*, 34*, 38*, 39*, 41*, 44*, 47*] of the 29 articles reported factors that either facilitated or impeded guideline implementation. A detailed overview of the characteristics of the included articles, along with their objectives and results concerning implementation outcomes, is presented in Table 1.

**Table 1:** Summarised characteristics of the included studies

**Author/year** **[Location]**	**Publication type**	**Setting (inpatient, outpatient)**	**Research aim**	**Study design**	**Population**	**Guideline**	Results regarding guideline implementation (*) **Barriers (-)/Facilitators (+)**	**Implementation outcomes**
Albizu-Rivera et al. 2015 [USA] [22*]	Original article	Inpatient cancer centres from the National Comprehensive Cancer Network (NCCN)	Assess implementation of key aspects of the palliative care (PC) guidelines by NCCN member institutions	Quantitative online survey	Institutional representatives from NCCN-member institutions (*n* = 21)	NCCN Palliative Care Guidelines	(*) Guideline recommendations compared with information provided by representatives from member institutions (*) Varying degree of implementation, with 81% of state PC performed in accordance with the guideline (*) Guideline primarily adopted `to inform clinical practice’, rarely to screen patients for PC needs	(2) adoption (5) fidelity/adherence
Brown et al. 2019 [USA] [23*]	Original article	Inpatient level 1 trauma centre	Integrate evidence-based PC for geriatric trauma patients in a multidisciplinary team	Retrospective chart review, quantitative post-implementation analysis	*n* = 188 geriatric trauma patients; (*n* = 94 pre- implementation, *n* = 94 post-implementation)	Palliative Care Best Practice Guideline (American College of Surgeons, 2017)	(*) After implementation: significant increase in the frequency of advanced care planning (ACP) directly after or before discharge; higher frequency of frailty assessments (*) Staff more willing to adopt ACP after education	(2) adoption (4) feasibility
Bush et al. 2022 [Canada] [38*]	Original article	Inpatient PC	Adapt, implement and evaluate a delirium guideline for a PC unit	Mixed-methods: online survey (quantitative), focus-groups/interviews (qualitative) and retrospective chart analysis	Staff of PC unit (*n* = 61) / PC unit patients (*n* = 40; *n* = 20 pre- implementation / *n* = 20 post- implementation)	Self-developed delirium guideline	(*) Education sessions were helpful, guideline was assessable, guideline was helpful for guiding delirium management, 72% intended to follow the guideline, some aspects from the guideline were already performed prior to implementation (*) Chart analysis showed guideline-adherent delirium management **(-) Barriers:** limited staff (e.g., night shift), complex symptoms, hierarchical differences, numerous changing circumstances in care provision, time constraints **(+) Facilitators:** protected time (‘time in which people can explicitly attend education’), common ‘language’ used by the entire team	(1) acceptability (3) appropriateness (4) feasibility (5) fidelity/adherence
de Putter et al. 2018 [Belgium] [42*]	Original article	Inpatient care	Evaluate current practice and compare it with international guidelines	Retrospective chart analysis	Cancer patients receiving palliative chemotherapy, and anaemia treatment (*n* = 72 episodes of care)	International Anemia guidelines: European Society for Medical Oncology (ESMO) & NCCN	(*) Medical documentation, compared with guideline recommendations (adapted to Belgian setting), in relation to diagnostics and therapy (*) Only approx. 50% patients treated according to the guidelines, also diagnostically (*) Adherent treatment could reduce consumption of blood products by up to 26% and impact patient quality of life (QoL)	(5) fidelity/adherence (6) penetration
Fasting et al. 2021 [Norway] [44*]	Original article	Outpatient / general practice	Investigate the adherence of GPs in Norway to the Norwegian Guideline for Palliative Care	Quantitative survey	GP in a northern Norwegian region (*n* = 142)	Norwegian guideline for Palliative care	(*) GP agreement on guideline content, information and use (*) Low guideline adherence, little adaptation of methods according to recommendations Paradox: recommended assessments seen as helpful, but not implemented ‘because it is not worthwhile with the small number of cases’ **(-) Barriers:** low number of PC patients per GP, guidelines more likely developed for cancer patients, may not suit GP patients (more frailty, dementia or organ failure)	(1) acceptability (3) appropriateness (5) fidelity/adherence
Glare et al. 2013 [USA) [24*]	Original article	Inpatient, gastro-oncology ward of a comprehensive cancer centre	Assess the feasibility and sustainability of implementing the screening and referral components of guidelines	Retrospective chart analysis and quantitative survey	Patients with gastrointestinal cancers (*n* = 229); nurses from the ward (*n* = 16)	NCCN Clinical Practice Guidelines in Oncology for Palliative Care (2009)	(*) Primarily feasibility and ‘sustainability’ of screening according to guidelines. Additional step: views of nurses and the effects on referral behaviour (*) Significantly more PC consults, initiated by the guideline; nurses agreed that guideline screening was quick, helpful and unobstructive to their clinical routines; implementation was quick and promoted early integration (*) ‘Sustainability’ was only analysed for a 3-month period during the project, not classified as an implementation outcome according to Petermann [18]	(1) acceptability (4) feasibility
Hakonsen et al. 2008 [Scotland] [45*]	Original article	Inpatient PC (hospital and hospice)	Audit current practice of pain management by applying the Medication Assessment Tool for Cancer Pain (MAT-CP) to adult cancer patients	Retrospective chart analysis	*n* = 192 patients (*n* = 56 in hospitals, *n* = 136 in hospice)	WHO: Cancer pain relief: with a guide to opioid availability (1996) & Scottish Intercollegiate Guidelines Network: Control of pain in patients with cancer (2000)	(*) Adherence measured via 37 MAP-CP items (*) Overall adherence: 75.9%; 37 MAP-CP criteria: 21 high adherence criteria (>75%), 7 intermediate adherence criteria (50–75%), 9 low adherence (<50%) (*) Improvements possible, especially for pain assessment; significantly greater adherence in hospices than hospitals (different priorities of care)	(4) feasibility (5) fidelity/adherence
Johnson et al. 2004 [USA] [25*]	Original article	Hospice care	Characterise hospice use of and attitudes towards written symptom management materials	Quantitative survey	Population-based PC research network (PoPCRN) representatives from each hospice (*n* = 78)	Variable, depending on the institution -> Identification of which guidelines are used at all	(*) Survey of which guidelines, pathways, etc. used in their hospice and for which symptoms; guideline copies should be forwarded to the scientists (*) 68% reported use of guidelines or pathways, most frequently for constipation, pain, nausea and anxiety (*) Documents showed that half were medication orders, not guidelines; evidence-based symptom management was rarely implemented; few guidelines published to date	(6) penetration
Jox et al. 2011 [Germany] [32*]	Original article	Inpatient, intensive care units	Assess guidelines’ outcome quality, including implementation	Prospective-longitudinal study, pre-post evaluation, semi-quantitative survey	Physicians and nurses (*n* = 448; pre- implementation *n* = 197; post-implementation *n* = 251)	Guideline of the University Hospital of Munich on decisions at the end-of-life: Changing treatment goals for critically ill and dying patients, including dealing with advanced care directives	(*) Level of awareness, comprehensibility, acceptance and impact on confidence in action; medical law knowledge was also ‘tested’ at the second test date (*) Pre-implementation: two-thirds would like to have a guideline; interest among senior physicians = lowest; need among assistant doctors and non-management nursing staff = greatest (*) Post-implementation: one-third knew the guideline content; one-third had heard of the guidelines, one-third had not; those who knew the guidelines felt more confident in their actions; effect greatest for assistant doctors; knowledge of medical law improved significantly	(1) acceptability (6) penetration
Kalies et al. 2017 [Germany] [33*]	Original article	PC in Germany	1) Evaluate the prevalence of critical attitudes and beliefs that could hinder implementation of the new guidelines; and 2) evaluate differences between professional groups	Quantitative online survey	Members from the German Association for Palliative Medicine (DGP), *n* = 1,031	S3-Leitlinie Palliativmedizin für Patient*innen mit einer nicht heilbaren Krebserkrankung (Version 1.0, 2015)	(*) Analysis of prior publication of the S3 guideline (*) Quality of the guideline questioned and doubts raised about its implementation (*) Profession had almost no impact, oncologists were more positive (presumably because they were more used to guideline work) (*) > 70% stated that the guideline was not always up to date, 40% doubted that guideline authors were independent, 57.6% (tended to) agree that it is difficult to change routines **(-) Barriers:** Scepticism towards the authors or their independence, guidelines experienced as a ‘loss of autonomy’ or a ‘cookbook’ that must be followed, doubt that routines can be changed, general stigma towards PC **(+) Facilitators:** Clarification that the guidelines do not provide laws but only ‘guidance’ Oncologists held more positive attitudes because they were ‘used to guidelines’	(1) acceptability (4) feasibility
Kalies et al. 2018 [Germany] [34*]	Original article	PC in Germany	Professional willingness to adopt existing recommendations concerning PC. Focus on differences between professions/settings	Quantitative online survey	Members from DGP *n* = 1,031	S3-Leitlinie Palliativmedizin für Patient*innen mit einer nicht heilbaren Krebserkrankung (Version 1.0, 2015)	(*) Three main barriers identified: lack of knowledge, lack of motivation and lack of outcome expectancy (*) Low knowledge: approx. 50% were unaware of existing recommendations; approx. 50% of those aware of the recommendations saw no improvement with implementation (*) Most guidelines only available in English (*) Gender and profession impacted motivation for implementation; female doctors: smallest lack of motivation; knowledge from nurses was lower **(-) Barriers:** lack of knowledge and lack of outcome expectancy **(+) Facilitators:** Profession – nurses felt less competent, and greater support for nurses could improve acceptance of future guidelines	(1) acceptability (4) feasibility
Kell et al. 2009 [United Kingdom] [36*]	Original article	PC / HIV in Lesotho	Explore whether nurses think that the World Health Organisation (WHO) Integrated Management of Adolescent and Adult Illnesses (IMAI) guidelines are a useful tool for the implementation and scaling-up of PC services	Qualitative study, semi-structured interviews	Nurses (*n* = 10) and key informants (*n* = 6) from two hospitals	WHO IMAI Guidelines	(*) Knowledge about the guidelines and the implementation of PC assessed; nurses asked whether they found the guidelines helpful (*) Poor knowledge about PC – PC guidelines/guidebooks were not used because they were considered ‘scary’ (*) Workshops/training had taken place; one training was not enough. Guideline training had a different focus (HIV therapy) and PC was only a background topic, which reduced the use of guidelines	(1) acceptability (2) adoption (3) appropriateness
Kim et al. 2020 [Canada] [39*]	Original article	Palliative radio oncology	Assess whether electronic dissemination of Choosing Wisely Canada (CWC) guidelines to radiooncologist led to increased use of single fraction radio therapy (SFRT)	Retrospective chart analysis	Patients treated with palliative radiotherapy for bone metastasis (*n* = 807)	CWC guideline for radiotherapy	(*) Guideline dissemination had no effect on care provision; large gap between known evidence (SFRT use recommended) and clinical behaviour (MFRT performed) (*) Guideline adoption challenging, especially when recommendations contradicted clinical routines **(-) Barriers:** Dissemination could be perceived as an external restriction of physician decision-making; within smaller organisations, scepticism could arise; dissemination by email considered unsuitable due to the high number of emails sent to oncologists	(5) fidelity/adherence (6) penetration
Koesel et al. 2019 [USA] [26*]	Original article	Outpatient PC	Patients: (a) receive guideline-based consultation evidenced by fidelity with standardised documentation, (b) rate their symptoms (i.e. pain, fatigue, anxiety) three times and (c) experience pain, fatigue and anxiety	Pre/post-test design, measurement of self-reported patient symptom scores related to pain, fatigue and anxiety	New patients with advanced cancer in two part-time PC clinics (*n* = 31)	American Society of Clinical oncology PC practice guideline (ASCO practice guidelines)	(*) Effect of guideline implementation on patient symptoms (pain, fatigue and anxiety) measured (*) Use of guideline-based procedures had a significant influence, with all symptoms significantly reduced between t1 and t3	(5) fidelity/adherence (6) penetration
LeBaron et al. 2021 [USA] [27*]	Original article	Inpatient cancer care institutions in Nepal	Design a mobile health application to scale up implementation of locally developed pain management guidelines	Cross sectional, quantitative survey study	Nurses (*n* = 64) and physicians (*n* = 28) from four hospitals	Nepalese Association of Palliative Care (NAPCare) Pain Management guideline (NAPCare PMG)	(*) Awareness and use of the NAPCare PMG questioned (*) 97% read the guideline, though nurses were significantly more likely to recognise its utility (*) 84% reported daily use (*) Use of the app was generally possible, as many used smartphones; the purpose was not necessarily recognised, aside from offering ‘education for patients and their families’	(1) acceptability (6) penetration
Lee et al. 2001 [United Kingdom] [20*]	Clinical audit	Inpatient care	Audit on oral care: implementing an oral care guideline	Clinical audit via questionnaire	Nurses from three wards from a hospital audit 1: *n* = 17 audit 2: *n* = 27	Selfmade: oral care guideline	(*) Following dissemination of the oral care guideline: 30-min training session, oral care information package (*) Guideline and training significantly improved oral care practice; previously untrained staff = high increase in knowledge, initially 20% good practice, then 56% (*) Significantly more (20% of 77% vs. 71% of 71%) patients who needed medication received it; training and guidelines improved oral care practice **(+) Facilitators:** when staff from the ward developed the guideline and conducted an audit, commitment increased and real changes were made in practice	(5) fidelity/adherence (6) penetration
Lind et al. 2017 [Sweden] [43*]	Original article	Inpatient PC / acute care	(1) Investigate perceptions of politicians, chief medical offers and health professionals regarding national PC guidelines; and (2) identify obstacles to and opportunities for implementation	Explorative qualitative interviews	Politicians (*n* = 6), chief medical officers (CMOs; *n* = 5) and health professionals (*n* = 29)	National Clinical Practice Guideline for Palliative Care; National Knowledge-Based Guide for Good Palliative Care in End-Of-Life Care	(*) Low knowledge across all disciplines, politicians, CMOs and staff (*) Politicians and CMOs emphasised the importance of guideline implementation, as patients had the right to equal care (*) No politician or CMO had ever read both guidelines (*) Staff mainly familiar with the short version and described a need for training; lack of time led staff to not prioritise dying patients	(1) acceptability (3) appropriateness (4) feasibility
Lovell et al. 2013 [Australia] [29*]	Original article	Specialist PC	Determine which guidelines for adult cancer pain are used and identify barriers to and facilitators of adult cancer pain guideline use	Cross-sectional quantitative online survey	PC physicians (*n* = 92)	Variable cancer pain guidelines -> Identification of which guidelines are used at all	(*) PC physicians asked which guidelines they used in relation to cancer pain (*) 45% of physicians routinely used one or more guideline on cancer pain, most commonly the ‘Therapeutic Guidelines Palliative Care, Version 3’ from the Australian PC expert group (*) All stated that guidelines work well, and 78% stated that their use influenced patient outcomes; guidelines were deemed necessary for the non-pharmacological management of cancer pain; cancer pain assessment and implementation strategies for existing guidelines were also rated as necessary	(1) acceptability (3) appropriateness (6) penetration
Lovell et al. 2022 [Australia] [30*]	Original article	Outpatient PC	Determine whether the guideline implementation strategy resulted in improved pain scores	Stepped wedge cluster randomised trial	Six centres in Australia, *n* = 754 patients (*n* = 359 control phase, *n* = 329 intervention phase)	Cancer pain guideline (not specified)	(*) Investigation of whether implementation strategies influenced patient pain levels: (1) audit of adherence to six key recommendations and feedback, (2) health professional education and (3) education booklet (*) No significant influences or differences between the intervention and control phases. No significant differences with regard to secondary outcomes such as QoL	(5) fidelity/adherence (6) penetration
Lo et al. 2019 [Singapore] [46*]	Original article	Different PC settings (hospitals, in-patient hospices and home care services)	Describe the national initiative to systematically develop and implement a set of national PC guidelines and quality measures	Mixed methods	healthcare professionals: standards development group (*n* = 9), expert panel (*n* = 14), guidelines implementation workgroup (*n* = 14), 11 PC services for case study (*n* = 220 patients)	National Guidelines for Palliative Care 2014 (NGPC)	(*) Description of the guideline development, explicit development of quality indicators (QI) from the guideline, retrospective examination of QIs for 20 patients from each of 11 PC services (*) Well-implemented pain assessment and documented care plans, as recommended (*) Opportunities for improvement identified (e.g., only 9% had response protocols for PC emergencies) (*) Voluntary audits well accepted by PC providers, use of guidelines could guide quality improvements well	(4) feasibility (5) fidelity/adherence
Lyon et al. 2007 [Australia] [21*]	Original article / evidence utilisation	Residential aged care	Ensure the ACP process is practiced according to the best available evidence-based guideline	Pre/post audit using Getting Research into Practice (GRIP) strategies	Residents from Manningham centre, residential care (*n* = 46)	Guidelines for a Palliative Approach in Residential Aged Care / ACP	(*) Prior to guideline implementation, recommendations not adopted, no ACP (*) Post-implementation, significant increase (not 100%, as participation in ACP was still voluntary and not all residents wanted to participate) **(-) Barriers:** lack of staff training / GP reluctance to participate **(+) Facilitators:** strong leadership fostered confidence that best practice could be achieved when guidelines were followed	(4) feasibility (5) fidelity/adherence
McIlfatrick et al. 2019 [United Kingdom] [37*]	Original article	Inpatient PC	Examine the clinical practice for the assessment management of constipation for patients with advanced cancer	Descriptive, retrospective chart analysis	Case notes from specialist PC (SPC) patients (*n* = 150)	Clinical guidelines for constipation (not specified)	(*) Variable, within and between wards (*) Holistic assessments, as required by the guideline, well implemented (*) Pharmacological interventions documented more frequently than non-pharmacological interventions (*) Nurses played a key role in the identification and treatment of constipation	(5) fidelity/adherence (6) penetration
Noble et al. 2018 [Australia] [31*]	Original article	Inpatient care	Investigate whether and how end-of-life (EoL) care excellence can be embedded or normalised in acute health care settings. Also, describe individual and contextual barriers and enablers surrounding implementation of the clinical guidelines for dying patients	Explanatory, qualitative interview study	Healthcare professionals (*n* = 28 nursing, medical and allied health professionals, PC team)	Clinical Guidelines for Dying Patients (CgDP)	(*) Normalisation process theory (NPT) approach, identified via (individual and group) interviews (*) HCP attitudes towards the guideline collected as barriers and facilitators. Many different influences on the guideline and its implementation were present in the acute setting **(-) Barriers:** EoL care = ‘failure’ in acute care, lack of education in EoL care provision, lack of multidisciplinary teamwork, lack of understanding roles related to the CgDp, other healthcare workers’ feelings of exclusion (due to a focus on nurses/physicians), paper-based documentation (as opposed to the typical electronical documentation) **(+) Facilitators:** Guideline signalled a shift towards a more structured and systematic approach to EoL care, emphasising the needs of dying patients in acute care settings, legitimising the provision of EoL care in such environments, empowering nurses to engage in meaningful discussions with medical staff (fostering a clear delineation of responsibilities) and promoting effective collaboration between nursing and medical teams, thereby enhancing continuity of care for patients	(1) acceptability (2) adoption (3) appropriateness
Noome et al. 2016 [Netherlands] [47*]	Original article	Intensive care	Examine the effectiveness of supporting intensive care units (ICUs) in implementing the guideline ‘End-of-life care in the ICU nursing care’	Cluster randomised controlled trial, mixed methods (questionnaire and interviews)	ICUs in the Netherlands (*n* = 16, *n* = 8 intervention, *n* = 8 control); interviews with nurses (*n* = 32); questionnaire: all nurses from participating ICUs (*n* = 265) and families of deceased patients (*n* = 33)	End-of-Life Care in the ICU, Nursing Care	(*) Intervention group received support programme when implementing the guideline, others not (*) Nurses trained as ‘implementation leaders’ could exchange ideas and discuss barriers and problems (*) Both control and intervention groups showed improved guideline adherence; intervention group demonstrated more positive effects, as they thought about the possibilities of patients dying at home and single room options significantly more often (*) Training provided only minimal benefit, and a structured implementation process was recommended (*) Patient families in the intervention group showed significantly greater satisfaction **(-) Barriers:** organisational/systemic aspects, lack of time to develop implementation strategies, difficulty coping with yet another innovation, large teams, difficulty reaching everyone, major organisational changes during implementation (e.g., merging or moving to a new building) **(+) Facilitators:** structured implementation process including education, audit and feedback, reminders, open-minded colleagues regarding EoL care, support from management, team implementation leaders of own station	(1) acceptability (5) fidelity/adherence
Pfister et al. 2010 [Switzerland] [48*]	Original article	Internal /general medicine and intensive care medicine	Better understand the impact of guidelines from the Swiss Academy of Medical Sciences (SAMS): Are physicians and nurses familiar with the guidelines, do they use them in daily practice and do they understand the legal status?	Quantitative survey	*n* = 843 respondents, no subgroups reported. Questionnaire 1: GPs, internists, nurses; Questionnaire 2: intensive care physicians, intensive care nurses	SAMS: Care of Patients in the End-of-Life (2004), Palliative Care (2006), Borderline Questions in Intensive Care Medicine (1999), The Determination of Death in the Context of Organ Transplantation (2005)	(*) Knowledge/attitudes of medical staff surveyed in two separate groups – palliative guidelines knowledge, use and legal status were surveyed among internists, nurses and GPs (Questionnaire 1) (*) Conclusion generalised for both groups; 80% had at least heard of the guidelines (*) Physicians more likely to know the guidelines than nurses; positive association between higher age and knowledge of each guideline (*) Younger individuals require greater consideration during the dissemination process, as they have high uncertainty about legal status	(3) appropriateness (6) penetration
Rojas-Concha et al. 2023 [Denmark] [40*]	Original article	Specialised PC	Investigate the degree of implementation of treatment guidelines in advanced cancer patients	Quantitative registry-based study	PC patients with advanced cancer in SPC services (*n* = 11,330 patients)	Four PC treatment guidelines for pain, dyspnoea, constipation and depression	(*) Register data analysis: patients treated according to guidelines (*) Patients treated according to guidelines increased significantly to approx. 90%, dropped again at project end (due to project fatigue) (*) Smallest number of patients treated according to guidelines was for depression (*) Implementation for all guidelines in over 90% of PCS, only less for depression (70%); implementation for physical symptoms better overall than implementation for psychic symptoms	(5) fidelity/adherence (6) penetration
Schubert et al. 2010 [Germany] [35*]	Original article	Outpatient, GP PC	Acceptance and implementation of the guideline recommendation should be determined within quality circles	Quantitative survey	GPs (*n* = 391)	Palliativversorgung (2007) Hausärztliche Leitliniengruppe Hessen)	(*) Guideline design rated positive (scope appropriate 75%, easy to use 74%, high practical relevance 71%, recommend 82%) (*) Pharmaco-therapeutic recommendations considered relevant, general high relevance (80–94%) and feasibility (75–91%) of recommendations (*) Only eight GPs stated that they had already implemented the guideline suggestions very well/good; high acceptance was no guarantee for implementation	(1) acceptability (2) adoption (3) appropriateness
Soerensen et al. 2023 [Denmark] [41*]	Original article	PC – inpatient clinical oncology/PC, outpatient general practice	Map barriers to and facilitators of the implementation of the national guideline in general PC for patients with incurable cancer	Qualitative descriptive study, semi-structured interviews	Healthcare professionals (*n* = 23)	Danish National Guideline on Palliative care (NG)	(*) Four years after publication, guideline still poorly implemented; in inpatient settings, nobody apart from the nurse manager had ever had contact with the guideline; implementation had only been supported by individual initiatives, if at all **(-) Barriers:** lack of knowledge, poor networking/lack of information exchange across sectors, guideline as ‘time burden’, uncertainty about incorporating the guideline into local guidelines, complicated relationships between patient and GP/oncologist, no structured plan for implementation, too lengthy **(+) Facilitators:** everyone shares common goal of fulfilling patient needs, motivation and competency to employ changes, guideline provides a common language, short form available, manager influences in a positive way, district nurses could help to improve knowledge	(1) acceptability (2) adoption (6) penetration
Vogel et al. 2020 [USA] [28*]	Original article	Inpatient geriatric trauma care	(1) Measure and compare satisfaction with PC before and after implementation of the American College of Surgeons Trauma Quality Improvement Program (ACS-TQIP) Palliative Care guidelines and (2) identify areas for quality improvement	Quantitative, prospective pre-post study	Patients < 55 years with trauma (*n* = 572; *n* = 299 pre, *n* = 273 post) primary caregiver, family member representative (*n* = 595; *n* = 334 pre- implementation,* n* = 261 post-implementation)	ACS-TQIP Palliative Care Best Practice Guidelines	(*) Implementation led to significantly greater patient satisfaction, especially in area of ‘information’ (*) No effects found for caregivers with already high satisfaction at T0 (*) Significantly lower satisfaction observed when patients/families received a prognostic assessment, reflecting a general challenge	(1) acceptability (3) appropriateness (6) penetration

### Descriptive analysis of topics

The descriptive analysis is divided into two parts. First, findings on the extent and effectiveness of guideline implementation are presented. Second, findings on the facilitators and barriers to guideline implementation are shown.

### Results for guideline implementation

Out of the eight implementation outcomes proposed by Petermann (2014), only six were considered in the review, as outcomes (7) ‘implementation costs’ and (8) ‘sustainability’ were not evaluated in any of the included studies. Regarding the outcome of sustainability, Petermann’s definition is formulated in a broad manner, encompassing the extent to which a newly implemented treatment is maintained or institutionalised. In the context of this review, cross-sectional studies that assess the implementation of guidelines within the context of research endeavours characterised by a limited duration are deemed ineligible for inclusion as studies evaluating sustainability. This is predicated on the premise that the experimental setting and the inherently brief nature of research projects do not accurately reflect the long-term utilisation of guidelines.

#### (1) Acceptability

Guideline acceptability was the most frequently investigated outcome, analysed in 15 articles [24*, 27*, 28*, 29*, 31*, 32*, 33*, 34*, 35*, 36*, 38*, 41*, 43*, 44*, 47*]. These studies examined the extent to which healthcare professionals perceived the guidelines and their content as satisfactory. Acceptability was exclusively investigated among healthcare professionals, with no studies addressing patient acceptance. Nurses, in particular – and to some extent assistant doctors – often viewed guidelines as helpful, to the point of explicitly requesting them [24*, 27*, 32*]. For example, Bush et al. [38*] found that a delirium management guideline on a palliative care ward was well accepted due to its perceived utility in facilitating healthcare work, with recommendations described as ‘well researched’. However, challenges to acceptability arose when healthcare professionals lacked knowledge about the guideline [34*, 36*, 43*]. Additionally, personal attitudes towards the guidelines (e.g., opinions held by professional societies/guideline groups) also influenced guideline development and implementation [33*].

#### (2) Adoption

Six of the articles [22*, 23*, 31*, 35*, 36*, 41*] analysed adoption, defined as the willingness to integrate guideline recommendations into clinical practice. This outcome was often addressed as a partial or secondary outcome rather than a primary focus. For example, Brown et al. [23*] reported a high willingness to adopt palliative care guidelines for geriatric trauma patients. In contrast, Kell et al. [36*] found that medical personnel in Lesotho deliberately disregarded palliative care guidelines/recommendations for HIV-positive patients due to fears and concerns surrounding the palliative care approach. Schubert et al. [35*] demonstrated that a positive evaluation of outcome (3) ‘appropriateness’ by general practitioners did not necessarily translate into practical guideline adoption.

#### (3) Appropriateness

Guideline appropriateness, defined by relevance, compatibility with user needs and timeliness, was examined in nine articles [28*, 29*, 31*, 35*, 36*, 38*, 43*, 44*, 48*]. In some cases, appropriateness overlapped with outcome (1) ‘acceptability’, as guidelines were frequently described as ‘helpful’ [38*], appropriately concise [35*], and ‘useful’ [44*] or ‘relevant’ [35*]. Lovell et al. [29*] found that palliative care professionals using cancer pain guidelines describe them as ‘working well’.

Another important finding related to the broader factors influencing successful implementation: for a guideline to be successfully implemented, it must not only be appropriate, but its training content and implementation strategies must align with user needs [36*]. To maximise the effectiveness of guideline training, researchers recommended that courses focus specifically on guideline content and avoid incorporating unrelated topics [36*]. Additionally, they highlighted that implementation strategies should include tailored dissemination efforts considering all relevant target groups. For instance, Pfister et al. [48*] identified a significant age-related disparity in guideline awareness, with younger physicians and nurses less likely to encounter the guidelines compared to their more experienced counterparts.

#### (4) Feasibility

Feasibility, defined as the extent to which guidelines could be practically implemented in a given setting, was addressed in nine articles [21*, 23*, 24*, 33*, 34*, 38*, 43*, 45*, 46*], with predominantly positive results. In research project settings, guidelines were often rated as easy to implement [24*, 38*] and ‘quick’ [21*] or ‘easy’ [38*] to introduce. However, challenges to feasibility were highlighted in two studies by Kalies et al. [33*, 34*], particularly regarding the difficulty of breaking established routines and introducing new behaviours into established care pathways. Language barriers were also identified as a challenge, particularly when guidelines or translations in the relevant national language were unavailable or difficult to access [33*, 34*]. Shortened or simplified versions of guidelines were viewed as particularly advantageous for practical implementation. Healthcare professionals generally reported that guideline implementation was feasible, provided it did not significantly disrupt the routine flow of clinical practice [24*, 43*].

#### (5) Fidelity/adherence

Fidelity (or adherence) to guidelines was analysed in 14 articles [20*, 21*, 22*, 26*, 30*, 37*, 38*, 39*, 40*, 42*, 44*, 45*, 46*, 47*]. The results indicated that a positive assessment of a single implementation outcome did not necessarily guarantee success in others. For example, Kim et al. [39*] found that the mere dissemination of guidelines did not ensure adherence. Similarly, Fasting et al. [44*] found that high levels of guideline acceptability (outcome 1) did not directly lead to greater adherence. Two quantitative studies reported substantial improvements in symptom management when care was delivered in accordance with the relevant guidelines [26*, 40*]. However, Rojas-Concha et al. [40*] highlighted variations in adherence, noting that adherence was generally higher for the management of physical symptoms (e.g., pain, dyspnoea) compared to psychological disorders (e.g., depression). Finally, Lovell et al. [30*] found that strategies developed for the implementation of pain management guidelines had no significant effect on fidelity.

#### (6) Penetration

Fourteen articles [20*, 25*, 26*, 27*, 28*, 29*, 30*, 32*, 37*, 39*, 40*, 41*, 42*, 48*] analysed guideline penetration, referring to their integration in healthcare settings. Similar to the findings reported for adherence (outcome 5), the results for penetration underscored that mere dissemination (e.g., via email) did not ensure penetration [39*]. Studies revealed that projects lacking in targeted implementation interventions tended to show low penetration [41*, 42*]. In contrast, penetration was typically higher during the active phases of implementation projects. However, in some cases, penetration declined towards the end of these projects, suggesting challenges in sustaining integration over time [40*]. Koesel et al. [26*] and Vogel et al. [28*] demonstrated that higher penetration led to significant improvements in patient outcomes and satisfaction.

Knowledge of guidelines among healthcare professionals was another critical factor influencing penetration. Johnson et al. [25*] found that more than two-thirds of hospice research network managers reported a use of guidelines, suggesting high penetration. However, an analysis of the corresponding ‘guideline’ documents revealed that over 50% failed to meet the formal definition of a guideline, thereby relativising the reported penetration level [25*].

### Facilitators of and barriers to guideline implementation

Facilitators of and barriers to the implementation of palliative care guidelines were explicitly or implicitly addressed in 10 [20*, 21*, 31*, 33*, 34*, 38*, 39*, 41*, 44*, 47*] of the 29 studies. Most of these studies detailed these factors in the results sections [21*, 22*, 31*, 33*, 34*, 38*, 41*, 47*], while three referenced facilitators and/or barriers only in the discussion sections, without further elaboration on implementation [20*, 39*, 44*]. The methods used to identify these factors varied: some studies employed surveys conducted prior to guideline implementation [33*, 34*], while others identified influencing factors post-implementation through interviews and surveys as part of the evaluation process [20*, 21*, 31*, 38*, 39*, 41*, 44*, 47*].

### Facilitators

Three articles identified a high level of motivation and willingness to embrace change as key facilitators [20*, 41*, 47*]. Noome et al. [47*] demonstrated that healthcare professionals who maintained an open mind and positive attitude towards change were more likely to support guideline implementation. Furthermore, motivation increased when members of the palliative care unit were actively involved in guideline development [20*] – an effect that was further enhanced when specific team members acted as ‘implementation leaders’, systematically encouraging adherence [47*]. Support from management was highlighted as a crucial facilitator in three articles [21*, 41*, 47*], with management’s favourable attitude towards guidelines [41*] and enactment of motivating leadership emphasising the positive effects of implementation [21*]. Moreover, the allocation of ‘protected time’ during working hours for guideline training was found to significantly improve knowledge and subsequent implementation [38*].

Short versions of guidelines were consistently viewed as helpful, as they were easier to reference during daily clinical practice compared to longer, more detailed versions [41*].

Two studies showed that guideline implementation was often rated positively by non-physician staff, and particularly nurses [31*, 34*]. Additionally, Kalies et al. [34*] demonstrated that guideline implementation enhanced competence among nurses and other non-physician staff, creating an empowering impact. Similarly, another study showed that guideline adherence enabled interdisciplinary discussion on clinical matters, fostering equality among healthcare professionals [31*]. This empowerment and inclusivity encouraged less experienced physicians and non-physician staff to engage actively, thereby promoting guideline implementation [31*, 34*]. Additionally, guideline implementation was shown to facilitate the creation of a ‘common language’ in palliative care settings, particularly for specific symptoms, ensuring consistent and effective communication among team members [38*, 41*].

Kalies et al. [33*] underlined the importance of healthcare professionals understanding the purpose of guidelines. Specifically, the authors found that guidelines must be perceived as recommendations, rather than rigid standards, alleviating concerns that they must be strictly followed.

In a Danish study, a national guideline for palliative care for cancer patients was examined with regard to its cross-sectoral implementation between outpatient and inpatient palliative care settings [41*]. The authors showed that implementation was facilitated by the involvement of experienced healthcare professionals with contact across all healthcare sectors (e.g., district nurses). These professionals acted as key figures who guided the process, monitored progress and addressed emerging challenges, thereby enhancing implementation success [41*].

### Barriers

Lack of time was frequently reported as a significant barrier to guideline implementation and use [38*, 41*, 47*]. For example, one study showed that the implementation process, itself, could require more time than was typically available in daily clinical practice [38*]. Moreover, insufficient time to develop appropriate implementation strategies was identified as a challenge to successful implementation [47*].

Another identified barrier was the need for changes in practice and setting. Interviews with nurses from intensive care units during the implementation of end-of-life care guidelines revealed that guideline application was particularly challenging when it required changes to clinical practice, especially in healthcare facilities undergoing larger organisational change [47*]. In particular, major changes, such as the relocation of wards or facilities to new buildings, were found to hinder implementation [47*]. Additionally, large teams (especially in shift-based systems) were identified as a barrier, making it more difficult to engage all members in the implementation process [47*]. Moreover, settings with few employees per shift (e.g., night shifts) tended to show less adherence to guidelines, particularly during the management of complex symptoms [38*].

Three articles showed that scepticism and lack of knowledge among healthcare professionals represent further barriers to guideline implementation [31*, 34*, 41*]. Scepticism was often accompanied by low outcome expectancy, which could negatively impact guideline implementation [34*]; and lack of knowledge was often attributed to insufficient staff training [21*, 41*].

Another significant challenge was observed when healthcare professionals failed to understand the purpose of a guideline or perceived it as obligatory [33*, 39*]. Two quantitative studies showed that the pressure to apply a guideline in every situation often reduced healthcare professionals’ sense of autonomy [39*] or made guidelines feel like ‘cookbook medicine’ [33*]. Such perceptions could result in scepticism and reluctance to engage with the recommendations.

In a qualitative framework study, Noble et al. [31*] examined clinical guidelines for dying patients and found that a failure to address all professional groups involved in care provision in implementation strategies represented a critical barrier. Most of the strategies and recommendations these authors reviewed focused on physicians and nurses, while overlooking other groups, such as therapeutic professionals [31*]. In addition, poor communication among team members [41*] and insufficient multidisciplinary collaboration [31*] were identified as barriers to successful implementation.

Sørensen et al. [41*] reported an additional challenge stemming from the concurrent use of regional and national guidelines. When healthcare professionals were uncertain about the manner in which regional guidelines aligned with the national ones, or when the guidelines conflicted, implementation of the national guidelines was often hindered.

Finally, Noble et al. [31*], in their interviews with healthcare professionals, identified specific barriers to the implementation of palliative care guidelines in acute care settings. Some professionals perceived the introduction of palliative care guidelines as a sign of failure in acute care. The authors also reported that lack of knowledge about palliative care principles acted as a barrier to successful guideline implementation [31*].

## Discussion

### Summary of the evidence

The present scoping review identified 29 heterogeneous studies that addressed the implementation of palliative care guidelines across six key dimensions: (1) acceptability, (2) adoption, (3) appropriateness, (4) feasibility, (5) fidelity/adherence and (6) penetration. Additionally, 10 studies reported on the barriers to and facilitators of guideline implementation.

#### Knowledge about the implementation of palliative care guidelines

This present findings highlight significant opportunities associated with the implementation of palliative care guidelines, particularly in achieving high levels of fidelity (outcome 5) and penetration (outcome 6). Studies demonstrated that, when guideline adherence and integration into health settings was high, symptom management and satisfaction among patients and families improved [26*, 28*, 47*].

Successful implementation was shown to require careful consideration of the context and setting, with the willingness and motivation of healthcare professionals to support implementation playing a critical role. The review therefore showed that guidelines were more effectively implemented when they caused only minimal disruption to existing care routines [24*, 35*, 38*].

The complexity of healthcare settings (often characterised by competing priorities) was found to present numerous barriers to successful implementation. In some of the included articles, guidelines were implemented and evaluated within the context of short-term research projects, frequently yielding positive outcomes [20*, 21*, 26*, 28*]. However, Rojas-Concha et al. [40*] showed that guideline penetration tended to decrease towards the end of the project period, due to ‘project fatigue’. These findings suggest that implementation within a controlled research setting may not correspond to real-world practice. For the sustainable implementation of guidelines, independent of time-limited interventions, broader considerations are necessary.

None of the reviewed articles investigated the implementation outcome of sustainability [18], possibly due to the inherent design of clinical guidelines, which are typically scheduled for periodic update [6, 49]. Update intervals vary by discipline and country (e.g., in Germany, the S3 guideline on palliative care for people with incurable cancer is updated every 5 years [13, 49]), and these predetermined time frames may leave only a brief window for implementation and sustainability evaluation. Thus, once an update occurs, new and modified recommendations must be promptly implemented and subsequently evaluated.

#### Knowledge about facilitators and barriers

Health professionals’ high levels of knowledge and expertise were shown to positively impact many implementation outcomes, including (1) acceptability, (2) adoption and (5) fidelity/adherence. In particular, training on guidelines and their content emerged as a key approach. However, the studies also demonstrated that successful guideline implementation requires more than just the provision of training; it also requires a willingness among healthcare professionals to adopt changes, which may be enhanced through support from ward or institutional management [38*, 41*, 47*]. Two systematic reviews of implementation barriers and facilitators across all medical specialties [50, 51] reinforced these findings, emphasising the importance of both guideline education and healthcare professionals’ motivation as facilitators.

The review also demonstrated that healthcare professionals’ commitment to guideline implementation tended to improve when the guidelines did not restrict their decision-making autonomy [33*, 39*]. Correa et al. [50], in their systematic meta-review of barriers and facilitators for the implementation of clinical practice guidelines, reported similar findings, also identifying barriers when guidelines were perceived as overly rigid or significantly conflicting with existing healthcare practices.

The identified facilitators and barriers are not exclusive to the palliative care context. Rather, many of the observed factors influencing guideline implementation (e.g., motivation and attitudes of healthcare professionals, management support, IT infrastructure, facilities) are applicable across various healthcare settings. In the reviewed articles, palliative care–specific factors were rarely reported, though one study suggested that palliative care, itself, may hinder guideline adoption (outcome 2) [31, 51], in their systematic meta-review of factors influencing healthcare professionals’ implementation of clinical guidelines, reached similar conclusions. Their findings confirmed key barriers such as a lack of time and insufficient management support, alongside facilitators such as effective implementation strategies and educational interventions tailored to the guidelines [51].

#### Implications for future research and practice

The finding that implementation outcomes can be achieved independently of one another carries important methodical implications for further research. Only three of the reviewed studies employed a mixed-method approach [38*, 46*, 47*], while the majority relied exclusively on either quantitative or qualitative methods. However, the evaluation of implementation outcomes, as proposed by Petermann [18], often requires a combination of methodological approaches. For example, outcomes (5) ‘fidelity/adherence’ and (6) ‘penetration’ are most effectively assessed using quantitative methods, while outcomes (1) ‘acceptability’ and (2) ‘adoption’ are more appropriately explored using qualitative interviews to capture participant attitudes. Thus, a mixed-method approach may be most suitable for evaluating all eight implementation outcomes, particularly when a comprehensive analysis of the implementation process is desired, addressing multiple dimensions.

Such research may provide valuable insights to inform practice, even prior to guideline implementation. The present review demonstrated the critical role of implementation strategies in the guideline development process. In Germany, the AWMF stipulates that implementation strategies must be explicitly delineated during guideline development [7]. The present findings indicate that implementation strategies should address as many implementation outcomes as possible, so the guidelines are not only accepted by healthcare professionals (outcome 1), but also practically feasible (outcome 4) and consistently adherent to (outcome 5), with wide penetration (outcome 6).

Similar findings were reported by a scoping review on the barriers to and facilitators of guideline implementation across various settings. In more detail, Fischer et al. [52] emphasised the importance of considering barriers and contextual factors at multiple levels during guideline development to create targeted implementation strategies promoting adoption and adherence. However, the review suggested that even well-designed implementation strategies may prove ineffective in practice, resulting in no discernible improvements in implementation. This should be carefully considered during the preparation of guideline implementation strategies [52].

The present review demonstrated that guideline dissemination should be tailored to the target audience prior to implementation – a conclusion that was also reached by Fischer et al. [52] in a separate scoping review. In particular, the present review highlighted the importance of considering younger healthcare professionals and non-physician staff, ensuring that dissemination strategies cater to their needs. Historically, non-physician staff have received limited attention during guideline development. However, recent years have seen a significant shift towards the inclusion of nurses and therapeutic professionals in the guideline development process [6]. Hence, dissemination strategies should aim to reach as many relevant healthcare professionals as possible, rather than targeting only subgroups or specialists within the care team.

Apart from the results regarding health care professionals, the review shows that few of the included studies assess patient-reported outcomes or outcomes pertaining to family caregivers. These findings are consistent with the results of Peters et al. [5], who examined trends in guideline implementation in a scoping review. Less than a quarter of their included studies examined patient-reported outcomes. Future studies of guideline implementation should therefore prioritise the examination of patient-reported outcomes to achieve a comprehensive evaluation of the effects of guideline implementation.

Regarding facilitators and barriers, the present review identified general influencing factors that were transferable across healthcare settings and aligned with those reported in other reviews and meta-reviews exploring other medical fields [50–52]. However, to enhance the success of future implementation projects, it may be beneficial to focus more closely on the specific contextual factors that may facilitate or hinder guideline implementation in palliative care, giving these more targeted attention.

#### Characteristics of the articles

Although some comparable findings were identified across the included studies, the overall results were highly heterogeneous. Most studies reported on standalone initiatives, thereby limiting the comparability and transferability of their outcomes. The studies analysed guidelines developed using various methodological approaches, covering diverse content areas and heterogeneous patient populations which should be taken into account when considering the transferability of the results, both for the Quince project and in general. The majority of the identified facilitators and barriers in the included studies are not specific to palliative care. Therefore, it can be assumed that they are at least comparable regardless of the guideline considered. Other reviews find comparable results, even when guidelines created for completely different settings and diseases are included [52]. Consequently, it can be assumed that these results are transferable and of further use in the Quincie project.

Additionally, a wide range of methods was employed, and different implementation outcomes assessed. The results indicate that, despite several years’ of research into the implementation of palliative care guidelines, the topic remains underexplored. Few studies were published prior to 2010 and, since then, there has been only a slight increase in the number of publications, consistent with the growing emphasis on guidelines and quality management in healthcare. This development highlights the need for more detailed evaluations of palliative care guidelines in future research [6].

### Limitations

The present scoping review faced challenges in defining the individual components of the research question. A key difficulty was the lack of a standardised international definition of the term ‘guideline’. Different terms, such as ‘guideline’ and ‘guidance’, are used internationally, and there is no consensus on the methodological or content-related criteria that a document must meet to qualify as a ‘guideline’ [53]. In the German context, for instance, guidelines are developed at varying levels of evidence [49]. It was not always evident from the reviewed articles whether the analysed documents were clinical care guidelines comparable to the S3 guideline for palliative care for patients with incurable cancer or whether the levels of evidence were equivalent, particularly in older studies. As a result, the findings may not be universally applicable to all national contexts. Similarly, the situation regarding implementation was characterised by considerable diversity, partly due to the variety of definitions for this term. The decision to systematise the outcomes using Petermann’s [18] taxonomy may have influenced the results. Consequently, the findings must be interpreted in light of the specific definitions of implementation outcomes. An alternative approach to organising the data, such as using the ERIC [54] or EPIS [55] framework, may have yielded diverging results and conclusions. Given the preliminary uncertainty regarding the scope of existing data concerning the implementation of guidelines in the field of palliative care prior to the review’s preparation, Petermann’s broad taxonomy of implementation outcomes was utilised [18]. Frameworks such as ERIC [54] meticulously delineate the components of implementation strategies, encompassing 73 items. In contrast, Petermann’s framework delineates a mere eight outcome dimensions [18]. Notwithstanding this discrepancy, the results of this scoping review evidence a degree of overlap among the outcomes. For example, the ERIC list [54] encompasses points such as ‘Distribute educational materials’, ‘Start a dissemination organisation’ and ‘Mandate change’, which are also found in the articles included in this review and relate to certain outcomes of Petermann’s model or could be identified as facilitators and barriers.

It is evident that guideline implementation in palliative care extends beyond the practical realisation of the recommendations, requiring consideration and evaluation of multiple implementation outcomes.

It is also possible that some relevant results were not included in this review due to the inclusion and exclusion criteria. In particular, this may apply to grey literature and articles not indexed in the selected databases.

## Conclusions

While individual projects have demonstrated both successful and less successful guideline implementation, the overall picture is highly heterogeneous. To gain a clearer understanding of the facilitators of and barriers to the implementation of palliative care guidelines, future research should aim at analysing these factors more comprehensively, giving particular attention to different national palliative care guidelines. In the implementation process, it is essential to consider a wide range of potential outcomes and to employ mixed-method designs for evaluation. In summary, the implementation of palliative care guidelines is a relatively nascent field of research that warrants further investigation.

## Supplementary Information

Additional file 1.

## Data Availability

The authors confirm that the data supporting the findings of this study are included in the article and its supplementary materials.
