# Supplementary material for: The implementation of guidelines in palliative care – a scoping review
Source: BMC Palliat Care. 2025 Apr 11;24:102. doi: 10.1186/s12904-025-01729-y (PMC11987174; doi:10.1186/s12904-025-01729-y)
Supplement: Supplementary file 1 — Additional file 1. [file 12904_2025_1729_MOESM1_ESM.docx]

| CINAHL (04.01.2024) |  |
| --- | --- |
| ((MW "palliative care" OR TI "palliative care" OR AB "palliative care" OR TI "terminal care" OR AB "terminal care" OR TI "palliative" OR AB "palliative" OR TI "terminally ill" OR AB "terminally ill" OR TI "terminal disease" OR AB "terminal disease" OR TI "end of life" OR AB "end of life" OR TI "eol" OR AB "eol" OR TI "lifes end" OR AB "lifes end" OR TI "advanced illness" OR AB "advanced illness" OR TI "advanced disease" OR AB "advanced disease" OR TI "end stage illness" OR AB "end stage illness" OR TI "life limiting illness" OR AB "life limiting illness") AND ( TI guidelines OR AB guidelines OR TI guideline OR AB guideline OR TI "quality indicators" OR AB "quality indicators") AND (TI implementation OR AB implementation OR TI implement OR AB implement OR TI "barriers and enablers" OR AB "barriers and enablers" OR TI "facilitating factors" OR AB "facilitating factors") NOT (TI "infant" OR AB "infant" OR TI baby OR AB baby OR TI babies OR AB babies OR TI "newborn*" OR AB "newborn*" OR TI "minor child" OR AB "minor child*" OR TI "dependent child*" OR AB "dependent child*" OR TI paediatric* OR AB "pediatric*" OR TI "paediatric*" OR AB "paediatric*" OR TI neonatal OR AB neonatal OR TI perinatal OR AB perinatal OR TI fetal OR AB fetal OR TI foetal OR AB foetal OR TI childhood OR AB childhood)) | **379 results** |
| LIVIO (04.01.2024) |  |
| (("palliative care"[MeSH Terms] OR "terminal care"[MeSH Terms] OR "terminal care"[Title/Abstract] OR "palliative"[Title/Abstract] OR "terminally ill"[Title/Abstract] OR "terminal illness"[Title/Abstract] OR "terminal illnesses"[Title/Abstract] OR "terminal disease"[Title/Abstract] OR "terminal diseases"[Title/Abstract] OR "end of life"[Title/Abstract] OR "eol"[Title/Abstract] OR "life's end"[Title/Abstract] OR "advanced illness"[Title/Abstract] OR "advanced illnesses"[Title/Abstract] OR "advanced disease"[Title/Abstract] OR "advanced diseases"[Title/Abstract] OR "end-stage illness"[Title/Abstract] OR "end-stage illnesses"[Title/Abstract] OR "end-stage disease"[Title/Abstract] OR "end-stage diseases"[Title/Abstract] OR "life-limiting illness"[Title/Abstract] OR "life-limiting illnesses"[Title/Abstract]) AND ("guidelines as topic"[MeSH Terms] OR "guidelines"[Title/Abstract] OR "guideline"[Title/Abstract] OR "quality indicators"[Title/Abstract]) AND ("implementation"[Title/Abstract] OR "implement"[Title/Abstract] OR "barriers and enablers"[Title/Abstract] OR "facilitating factors"[Title/Abstract]) NOT ("infant"[Title/Abstract] OR "infants"[Title/Abstract] OR "baby"[Title/Abstract] OR "babies"[Title/Abstract] OR "newborn"[Title/Abstract] OR "newborns"[Title/Abstract] OR "minor child"[Title/Abstract] OR "minor children"[Title/Abstract] OR "dependent child"[Title/Abstract] OR "dependent children"[Title/Abstract] OR "pediatric"[Title/Abstract] OR "pediatrics"[Title/Abstract] OR "paediatric"[Title/Abstract] OR "paediatrics"[Title/Abstract] OR "neonatal"[Title/Abstract] OR "perinatal"[Title/Abstract] OR "fetal"[Title/Abstract] OR "foetal"[Title/Abstract] OR "childhood"[Title/Abstract])) | **51 results** |
| PubMed (04.01.2024) |  |
| (("palliative care"[MeSH Terms] OR "terminal care"[MeSH Terms] OR "terminal care"[Title/Abstract] OR "palliative"[Title/Abstract] OR "terminally ill"[Title/Abstract] OR "terminal illness"[Title/Abstract] OR "terminal illnesses"[Title/Abstract] OR "terminal disease"[Title/Abstract] OR "terminal diseases"[Title/Abstract] OR "end of life"[Title/Abstract] OR "eol"[Title/Abstract] OR "life's end"[Title/Abstract] OR "advanced illness"[Title/Abstract] OR "advanced illnesses"[Title/Abstract] OR "advanced disease"[Title/Abstract] OR "advanced diseases"[Title/Abstract] OR "end-stage illness"[Title/Abstract] OR "end-stage illnesses"[Title/Abstract] OR "end-stage disease"[Title/Abstract] OR "end-stage diseases"[Title/Abstract] OR "life-limiting illness"[Title/Abstract] OR "life-limiting illnesses"[Title/Abstract]) AND ("guidelines as topic"[MeSH Terms] OR "guidelines"[Title/Abstract] OR "guideline"[Title/Abstract] OR "quality indicators"[Title/Abstract]) AND ("implementation"[Title/Abstract] OR "implement"[Title/Abstract] OR "barriers and enablers"[Title/Abstract] OR "facilitating factors"[Title/Abstract]) NOT ("infant"[Title/Abstract] OR "infants"[Title/Abstract] OR "baby"[Title/Abstract] OR "babies"[Title/Abstract] OR "newborn"[Title/Abstract] OR "newborns"[Title/Abstract] OR "minor child"[Title/Abstract] OR "minor children"[Title/Abstract] OR "dependent child"[Title/Abstract] OR "dependent children"[Title/Abstract] OR "pediatric"[Title/Abstract] OR "pediatrics"[Title/Abstract] OR "paediatric"[Title/Abstract] OR "paediatrics"[Title/Abstract] OR "neonatal"[Title/Abstract] OR "perinatal"[Title/Abstract] OR "fetal"[Title/Abstract] OR "foetal"[Title/Abstract] OR "childhood"[Title/Abstract])) | **766 results** |
| Web of Science (04.01.2024) |  |
| (TS=("palliative care" OR "terminal care" OR "palliative" OR "terminally ill" OR "terminal disease" OR "end of life" OR eol OR "lifes end" OR "advanced illness" OR "advanced disease" OR "end stage illness" OR "life limiting illness") AND TS=(guidelines OR guideline OR "quality indicators") AND TS=(implementation OR implement OR "barriers and enablers" OR "facilitating factors") NOT TS=("infant$" OR baby OR babies OR "newborn$" OR "minor child*" OR "dependent child*" OR "pediatric$" OR "paediatric$" OR neonatal OR perinatal OR fetal OR foetal OR childhood)) | **890 results** |
